# Diagnostic accuracy of ultrasonography in detection of blunt abdominal trauma and comparison of early and late ultrasonography 24 hours after trauma

**DOI:** 10.12669/pjms.314.6614

**Published:** 2015

**Authors:** Ali Feyzi, Masoud Pezeshki Rad, Navid Ahanchi, Jalil Firoozabadi

**Affiliations:** 1 Ali Feyzi, Assistant Professor of Radiology, Department of radiology, School of Medicine, Mashhad University of Medical Sciences, Mashhad, Iran; 2 Masoud Pezeshki Rad, Associate Professor of Radiology, Department of radiology, School of Medicine, Mashhad University of Medical Sciences, Mashhad, Iran; 3 Navid Ahanchi, Residency students of Radiology, Department of radiology, School of Medicine, Mashhad University of Medical Sciences, Mashhad, Iran; 4 Jalil Firoozabadi, Residency students of Radiology, Department of radiology, School of Medicine, Mashhad University of Medical Sciences, Mashhad, Iran

**Keywords:** Blunt abdominal trauma, Ultrasound Scan

## Abstract

**Objective:**

Despite the advantages of ultrasound scan, its use as a screening tool in blunt abdominal trauma is controversial. The aim of this study was to evaluate the diagnostic value of early and late ultrasound in patients with blunt abdominal trauma (BAT).

**Methods:**

In this study which was performed in a level I trauma center, firstly, 2418 patients with BAT had ultrasound (US) examination by two trauma expert radiologists. Results were compared with the best available gold standards such as laparotomy, CT, repeated ultrasound or clinical course follow-up. Then, 400 patients with BAT were examined by a trained residency student.

**Results:**

In the first phase, sensitivity, specificity, negative predictive value, positive predictive value and accuracy of ultrasound were 97%, 98.1%, 99.7%, 83% and 98% respectively. In the second phase, they were 97.3%, 97.2%, 97.7%, 96.8% and 97.3% for the early and 98.5%, 97.6%, 98.5%, 97.5% and 98% for the late ultrasound respectively.

**Conclusion:**

Results obtained from this study indicate that negative ultrasound findings associated with negative clinical observation virtually exclude abdominal injury, and confirmation by performing other tests is unnecessary. High sensitivity and negative predictive value is achieved if ultrasound is performed by expert trauma radiologist.

## INTRODUCTION

Trauma has become a significant health problem in part due to high velocity transportation. Improvements for those affected with trauma include faster rescue, better organization of trauma centers and advances in treatment. There are three recent trends in this regard: increasing tendency for non-operative care, the need for accurate non-invasive imaging diagnosis and a desire for cost-effective use of imaging. An aspect of the trend towards non-operative care is in favor of avoiding non-therapeutic surgery; this will be possible if imaging can identify those patients who require surgery.

Using Computed Tomography (CT) or ultrasound for detection of blunt abdominal trauma (BAT) injuries has its own advantages and disadvantages.^1^-^3^ CT has been shown to have the best statistical accuracy for detecting, characterizing and excluding injuries. However, it may be overused – in one study only three out of 100 patients had an alteration in clinical management after follow-up CT.^4^ There has been a lot of controversy over the use of ultrasound (US) as screening tool for detection of intra-abdominal fluid and organ injuries.^1^,^2^,^5^-^22^ Ultrasound operator’s skill and technique can largely affect the results. It seems that weak results obtained from some studies may originate from this fact. In our study, we aimed to evaluate diagnostic value of US when it is done by an expert radiologist in the field of trauma. In the second phase of our study, the same procedure was repeated by trained residency students of radiology.

## METHODS

This research was performed during two completely separate phases. First phase was performed during a period of 3 years on patients admitted in the Hashemi Nejad level I traumatological hospital in Mashad, Iran. Inclusion criteria defined to enroll all hemodynamically stable patients suspected of blunt abdominal trauma according to physician’s (and/or surgeon’s) decision who had at least one ultrasound request. The exclusion criteria included patients with hemodynamic instability, pregnant patients, end-stage renal disease, cases with abdominal inflammatory processes, patients with subcutaneous emphysema in whom abdominal ultrasound was difficult to interpret and patients without any clinical outcome.

All ultrasound examinations were performed by using a Siemens Sonoline G20 (Siemens, Germany) or a portable EUB-405 ultrasound instrument (Hitachi Medical, Japan). Also, all ultrasonographic examinations were done by only two experienced radiologists who were practically trained at least for 6 months in trauma radiology prior to the study.

To determine specificity and sensitivity of ultrasound, for each patient, results from the first ultrasound were compared with the best available standards for reference including surgical results, CT scan results, follow up ultrasound results as well as clinical follow up results.

In the second phase of the study, concerning 98% confidence coefficient and 92% volume power, during 4 months, 400 patients were introduced to the study in the same way as the previous phase. Two radiology specialty students were practically trained by radiologists in previous phase for 3 months and then, all ultrasonographic examinations of this phase were performed by these residents with the same instruments. Also, in this phase, all cases underwent at least two ultrasound examinations: early and 24 hour after trauma.

## RESULTS

In the first phase of the study, a total of 2415 patients suspected to BAT were studied. 127 cases underwent more than one ultrasound scans for follow-up purposes. In 2179 cases, first US were normal, reporting pathologies not related to trauma (such as renal stone, ovarian cyst, *etc*.) or reporting normal abdomen but suggestive of extra-abdominal trauma.

### First ultrasound report with no evidence of BAT

Among 2179 cases without US evidence of BAT, one patient underwent surgery whose abdominal organs were normal in laparotomy. Omental vein’s ligation surgery was done for this patient. 2 cases with normal ultrasound report were further studied by CT scan, which showed normal abdomen. One case experienced a deep coma and died after 4 days of admission due to brain injury without the need for abdominal intervention. For this patient, ultrasound was requested two times with 2 days interval which both were normal. Another patient who died had also two requests for ultrasound with 3 days interval, with both ultrasound reports indicating hepatic hydatidcyst. This patient died due to nonsurgical causes and without any further requests for CT or surgery.

Thirteen cases had repeated ultrasound requests during the next days. In 10 cases, follow-up ultrasound were normal or suggested pathologies other than BAT. In 3 cases, follow-up ultrasound showed evidences suggesting mild BAT (very low free fluid or mild organ injuries). Fluid which was observed in the 2^nd^ ultrasound reports was considered as blood; because the patients were males without any evidences for underlying conditions causing ascites.

### First ultrasound report indicating BAT

Among 236 patients with ultrasond evidence of BAT, 31 underwent surgery. In 30 patients, abdominal trauma was observed in laparotomy (30 True Positive (TP)). One patient had no evidence for abdominal trauma in laparotomy. Small spleen contusion (2×3 cm) without free peritoneal fluid was reported in ultrasound report for this patient. This case was labeled as False Positive (FP) result.

Four patients were followed up by CT-scan. CT results confirmed ultrasound reports completely (4 TP). Three patients were followed up by repeated ultrasound scans, which all suggested BAT and CT confirmed those results. One patient with positive BAT resulted in two repeated ultrasound and CT without the need for surgical intervention.

Eighty six cases had ultrasound reports indicating mild injuries in the abdomen. These patients underwent conservative treatment and their outcome suggested gradual relief. Finally, all of them were discharged with a good state of general health (86 TP).

Ninety seven cases with mild-BAT in ultrasound report were followed up by further ultrasound scans. 61 had second ultrasound suggesting mild injuries (61 TP) and in 36 cases, the second ultrasound was normal. These 36 cases were considered FP (even though there was a mean 4 days interval between the two reports, and the second ultrasound was requested for follow up and not for confirmation of the 1^st^ ultrasound). Non-mild BAT was conventionally defined as presence of free fluid in two or more regions in abdominal cavity.

Eighteen cases had non-mildBAT evidences in the 1^st^ ultrasound report. They were conservatively treated, followed up by further ultrasound examinations and were finally discharged from the hospital with a good general health state. Of these 18 cases, 15 showed mild injuries in the second ultrasound examination (15 TP) and 3 showed normal ultrasound (3 FP, the interval between two ultrasound reports was: 1, 3 and 6 days).

In the second phase of the study, results were analyzed for both the 1^st^ ultrasound and the 2^nd^ ultrasound similar to the first phase. Diagnostic values for both phases of the study are shown in Table-I.

**Table-I:** Diagnostic values obtained from phase I and II of the study.

	Phase I	Phase II	
		Early US	Late US
True Positive (TP)	196	179	192
True Negative(TN)	2173	210	200
False Positive(FP)	40	6	5
False Negative(FN)	6	5	3
Sensitivity	97%	97%	98%
Specificity	98%,	97%	98%
Positive predictive value	83%	97%	97%
Negative predictive value	99%	98%	99%
Accuracy	98%	97%	98%

## DISCUSSION

There is controversy about the appropriate use of ultrasound in the screening of blunt abdominal trauma. The main goal of screening imaging in blunt abdominal trauma is rapid identification of “life-threatening injuries” to enable treatment of the injuries in the initial hour after presentation of the patient to the hospital. Therefore, early identification of these cases is more important than a detailed evaluation of all injuries.

Although minor parenchymal damages in abdominal organs observed in CT scan may not be visible in ultrasound; but, it is important to note that in the vast majority of cases, invisible minor parenchymal injuries without concomitant free fluid, does not require surgery, and lower sensitivity of ultrasound would not adversely affect the patient’s clinical outcome.

Weak results for ultrasound obtained from some previous researches may originate from the role of operator’s skill in trauma ultrasonography and in some cases, from low number of enrolled patients (even as low as 34). In most previous studies, ultrasound has been performed by surgeons, emergency physician ultrasonographers or radiology residents with limited skills in this field. This is overshadowed by very large numbers of involved ultrasonographers with different skills. Also, in many trauma centers, FAST (Focused Abdominal Sonography for Trauma) is used as routine diagnostic procedure for the detection of intraperitoneal hemorrhage. These centers have reported to have false negative rate greater than 15%.^9^,^13^,^15^,^16^,^18^-^23^

Compared to other studies, our research benefited greatly from large volume of patients (all patients with BAT who were admitted to our trauma center during 3 years) and benefited from that all ultrasound examinations were done in each phase, by only two experienced radiologists using two ultrasound machines. During the study, mentioned radiologists were available round the clock and they performed all the processes of ultrasound actively (real-time examination, imaging and reporting). Also, instead of FAST, a “full potential” abdominal ultrasound examination was performed for all patients including examination of organ injuries. The second phase of our study clearly shows that the same results will be obtained if we accurately train sonologists.

In the first phase, 30 out of all 31 patients who underwent surgery also had an ultrasound indicative of abdominal lesion. Laparotomy in the only patient which was considered as False Negative ultrasound result suggested no abdominal organ injury and indicated bleeding from omental vessels. Therefore, the sensitivity in detection of abdominal injury in surgical patients was equal to 97%.

Another specific application of ultrasound which was defined in this study is the use of ultrasound for monitoring the patients who were candidates for supportive non-surgical treatment despite visceral lesion in their ultrasound. In this study, 115 patients with visceral injuries have been accurately monitored by serial ultrasound scans (even up to 8 times). This exclusive feature of ultrasound cannot be applied in other diagnostic methods such as Diagnostic Peritoneal Lavage (DPL) and CT scan.

Finally, we believe that due to very high sensitivity and negative predictive value, ultrasound can be used as a suitable tool for screening patients with blunt abdominal trauma, if performed by a radiologist experienced in the field of trauma. Normal ultrasound along with negative clinical examination is able to rule out the possibility of abdominal lesion and eliminate the need of further diagnostic measures.
